# A novel long non-coding RNA from the *HOXA6-HOXA5* locus facilitates colon cancer cell growth

**DOI:** 10.1186/s12885-019-5715-0

**Published:** 2019-06-03

**Authors:** Saki Saijo, Yuki Kuwano, Shoichiro Tange, Kazuhito Rokutan, Kensei Nishida

**Affiliations:** 1 Department of Pathophysiology, Institute of Biomedical Sciences, Tokushima University Graduate School, 3-18-15 Kuramoto-cho, Tokushima, 770-8503 Japan; 2 Department of Human Genetics, Institute of Biomedical Sciences, Tokushima University Graduate School, 3-18-15 Kuramoto-cho, Tokushima, 770-8503 Japan

**Keywords:** *HOXA5*, Non-coding RNA, Cell proliferation, Colon cancer, Epidermal growth factor signaling

## Abstract

**Background:**

Homeobox A5 (HOXA5), a member of the HOX family, plays an important role in tumor development and morphogenesis, although opposite effects on tumorigenesis have been observed, depending on the tissue type. In this study, we aimed to investigate the role of a novel transcript from the *HOXA6*-*HOXA5* locus in colon cancer tumorigenesis.

**Methods:**

Human colon cancer cell lines were analyzed using next generation sequencing-based targeted mRNA capture. The effects of overexpression and silencing of *HOXA5* transcripts were evaluated in vitro and using a xenograft nude mouse model.

**Results:**

We identified three novel transcripts (*HOXA5* short, long 1, and long 2) transcribed from the *HOXA6*-*HOXA5* locus in HCT116 colon cancer cells using next generation sequencing-based targeted mRNA capture. Knockdown of *HOXA5* long 1 and long 2 transcripts did not affect cell growth, while selective silencing of *HOXA5* short RNA inhibited cell growth independent of HOXA5 expression. Stable overexpression of *HOXA5* short RNA promoted proliferation and migration of colon cancer cell lines HCT116, DLD1, and HT-29 and accelerated tumor growth in the xenograft mouse model. In vitro translation assays suggested *HOXA5* short RNA was a functional long non-coding RNA (lncRNA). Consistent with these observations, expression of *HOXA5* short RNA was upregulated in advanced colon cancer tissues. Ingenuity Pathway Analysis of differentially expressed genes between *HOXA5* short RNA overexpressed and silenced HCT116 cells revealed that *HOXA5* short RNA preferentially modified expression of epidermal growth factor (EGF) signal-related genes. Western blot analysis demonstrated that stable overexpression of *HOXA5* short RNA increased EGF receptor levels and facilitated its phosphorylation in both HCT116 cells and xenograft tumors.

**Conclusions:**

Our results suggested that *HOXA5* short RNA, a novel lncRNA, may play a crucial role in colon tumor growth through activation of EGF signaling.

**Electronic supplementary material:**

The online version of this article (10.1186/s12885-019-5715-0) contains supplementary material, which is available to authorized users.

## Background

The *homeobox* (*HOX*) gene family, a large group of comparable genes, contains a common 183-nucleotide sequence (homeobox) that encodes a highly conserved 61-amino acid motif (homeodomain). The 39 members of the mammalian *HOX* gene family are organized into four clusters (*HOXA*, *HOXB*, *HOXC*, and *HOXD*) located on four different chromosomes and play a central role in the formation of body segment-specific structures through transcriptional regulation of downstream effectors during embryonic development [1]. The ability of *HOX* genes to control morphogenesis suggests they have essential roles in multiple cellular processes. Dysregulated expression of *HOX* genes is associated with oncogenesis and several lines of evidence indicated a potential role of *HOX* genes in tumor development of various tissues [2, 3].

HOXA5 plays crucial roles in embryo development and cell differentiation, especially in the respiratory system. Loss of HOXA5 function results in neonatal death due to respiratory distress [4]. Previous studies have revealed dysregulated HOXA5 expression in several cancers. HOXA5 is upregulated in oral squamous cell carcinoma [5] and its loss inhibits proliferation and cell tumorigenesis in esophageal squamous cell cancer [6] and acute myeloid leukemia cells [7], suggesting that HOXA5 may act as an oncoprotein in these cells. In contrast, HOXA5 expression is absent in several breast cancer cell lines and mammary carcinomas, and its absence correlated with higher pathological grades [8]. In a human colon cancer dataset, HOXA5 expression was lower in carcinomas compared with that in normal colon tissues, and high levels of HOXA5 expression was a prognostic factor for predicting improved relapse-free survival [9, 10]. A recent study showed that HOXA5 promoted differentiation by downregulating WNT signaling in colon epithelial cells and acted as a tumor suppressor in colon cancer tissues [9]. Thus, the functional significance of HOXA5 in tumor development and progression is likely dependent on the type of cancer cells involved.

It is clear that a large proportion of eukaryotic protein-coding genes (nearly 50% in human) serve as host genes for non-coding regulatory RNAs including small nucleolar RNAs, microRNAs, and long non-coding RNAs (lncRNAs) [11]. The *HOX* gene loci are particularly rich in lncRNAs, which may contribute to temporally and spatially restricted patterns of *HOX* gene expression throughout development. Several alternative transcripts are embedded as lncRNAs in the *Hoxa5* and *Hoxa6* locus of the mouse embryo [12].

Here, we identified a novel lncRNA (named *HOXA5* short RNA) transcribed from the *HOXA6*-*HOXA5* locus in human colon cancer cells using a next generation sequencing-based RNA capture system. Subsequent in vitro and in vivo experiments uncovered its oncogenic functions*,* providing new insight in the clinical relevance of *HOXA5* short RNA in tumorigenesis.

## Methods

### Cell culture

Human colon cancer cell lines HCT116 (CCL-247; American Type Culture Collection (ATCC), Manassas, VA), DLD1 (CCL-221; ATCC), and HT-29 (HTB-38; ATCC) were cultured in Dulbecco’s Modified Eagle Medium (DMEM; Nacalai Tesque, Kyoto, Japan) supplemented with 10% (vol/vol) heat-inactivated fetal bovine serum (FBS). Human lung cell lines A549 (CCL-185; ATCC) and BEAS-2B (CRL-9609; ATCC) were cultured in RPMI-1640 medium (Nacalai Tesque) supplemented with 10% FBS. Human colonic epithelial cells (HCEC-1CT) were obtained in December 2018 from Evercyte (Vienna, Austria) [13]. The cells were maintained in 4:1 DMEM (Nacalai Tesque)/Medium 199 (Gibco, Grand Island, NY), supplemented with 4 mM GlutaMAX (Gibco), 2% Cosmic Calf serum (Hyclone; Waltham, MA), 20 ng/mL human epidermal growth factor (PeproTech, Rocky Hill, NJ), 10 μg/mL insulin (Gibco), 2 μg/mL apo-transferrin (Sigma, St Louis, MO), 5 nM sodium selenite (Sigma) and 1 μg/mL hydrocortisone (Sigma). All cells were cultured at 37 °C in 5% CO_2_. All cell lines were routinely tested negative for mycoplasma contamination using the MycoAlert™ Mycoplasma Detection Kit (Lonza, Basel, Switzerland). To confirm HCT116 cells identity, short tandem repeat typing with GenePrint System (Promega, Madison, WI, USA) was performed in September 2014 and verified against the STR database of Japanese Collection of Research Bioresources.

### Next-generation sequencing (NGS)

Total RNA was extracted and purified from HCT116 cells using an RNeasy Mini Kit (QIAGEN, Hilden, Germany). To obtain *HOXA5*-enriched RNA, target-specific biotinylated DNA probes were designed, including *HOXA5* 3′ untranslated region (UTR)-targeted antisense sequences (probes 1 to 3) and *HOXA5*-*HOXA6* intergenic region-targeted antisense sequences (probes 4 to 6). Specific sequence details for the probes are provided in Additional file 1: Table S1. One microgram of total RNA was hybridized with mixed probes A (probes 1 to 3) or mixed probes B (probes 4 to 6) and the RNA-DNA complexes were then purified using AMPure XP beads (Beckman Coulter, Brea, CA, USA). The purified RNA-DNA complexes were captured using Capture Beads (Clontech Laboratories, Mountain View, CA, USA) and were subjected to reverse transcription and double stranded-complementary DNA (cDNA) amplification with SMARTer Target RNA Capture for Illumina (Clontech Laboratories). Double stranded-cDNAs were fragmented by an ultrasonic sonicator (Branson, Stamford, CT, USA). The fragmented DNA was tagged and amplified using the NEBNext Ultra DNA Library Prep Kit for Illumina (New England BioLabs, Beverly, MA, USA). Sequence reads encompassing *HOXA5* coding sequences were performed using a MiSeq system (Illumina, San Diego, CA, USA). The sequence data were quality-filtered using Trimmomatic 0.38 [14] and were mapped to the human reference genome assembly GRCh38 using STAR program (https://github.com/alexdobin/STAR). The mapped reads were further visualized using Integrative Genomics Viewer (IGV).

### Rapid amplification of cDNA ends (RACE) analyses

Total RNA was isolated from HCT116 cells and subjected to 5′-RACE and 3′-RACE analyses using a SMARTer RACE 5′/3′ Kit (Clontech Laboratories) according to the manufacturer’s protocol. Reverse transcription was performed using Powerscript reverse transcriptase with either the 5′-RACE cDNA synthesis primer or 3′-RACE cDNA synthesis primer. Details regarding the gene-specific reverse primers for 5′-RACE and gene-specific forward primers for 3′-RACE are listed in Additional file 1: Table S1. The polymerase chain reaction (PCR) products were gel purified and cloned into the pcDNA3 vector (Invitrogen, San Diego, CA, USA), and transformed into *E. coli* DH5a cells (Toyobo, Osaka, Japan) for Sanger sequencing.

### RNA interference and rescue experiments

Eight different small interfering RNAs (siRNAs; QIAGEN or Dharmacon, Lafayette, CO, USA) were used, each targeting different sequences within the respective *HOXA5* transcripts (Additional file 1: Table S1 and Additional file 2: Figure S1). AllStars Negative Control siRNA (QIAGEN) or ON-TARGETplus Non-targeting Control siRNA (Dharmacon) was used as control siRNA. HCT116 cells were treated with the indicated siRNAs at a final concentration of 10 nM using Lipofectamine RNAiMax (Invitrogen) according to the manufacturer’s instructions. For the rescue experiments, after HCT116 cells were treated with *HOXA5* siRNA #2 or control siRNA for 24 h, a plasmid containing HOXA5-encoding cDNA was transfected into the HCT116 cells using X-tremeGENE HP DNA Transfection Reagent (Roche Diagnostics, Indianapolis, IN, USA) according to the manufacturer’s instructions.

### Quantitative real-time reverse transcription-PCR (qPCR)

Total RNA was extracted using RNAiso Plus (Takara Otsu, Japan). One microgram of isolated RNA was reverse-transcribed using ReverTra Ace reverse transcriptase with genomic DNA (gDNA) Remover (Toyobo). *HOXA5* mRNA, *HOXA5* short RNA, and *HOXA5* long RNA levels were measured by qPCR using Power SYBR Green PCR Master Mix (Applied Biosystems, Foster City, CA, USA) and a 7500 Real-Time PCR System (Applied Biosystems). *Glyceraldehyde 3-phosphate dehydrogenase* (*GAPDH*) mRNA was used as an internal control for normalization. Data were analyzed using the ΔΔCt method. All primers used for qPCR are listed in Additional file 1: Table S1 and Additional file 2: Figure S1.

### Cell fractionation and western blotting

Whole-cell lysates and cytoplasmic or nuclear fractions were prepared using a Subcellular Protein Fractionation Kit for Cultured Cells (Thermo Scientific, Rockford, IL, USA). Xenograft tumor tissues were lysed for 30 min on ice in RIPA lysis buffer (Cell Signaling Technology, Beverly, MA, USA) supplemented with the protease inhibitor (Nacalai Tesque) and phosphatase inhibitor (Sigma–Aldrich, St. Louis, MO, USA). After centrifugation at 10,000×g for 15 min at 4 °C the supernatants were collected. The extracted proteins were separated by sodium dodecyl sulfate-polyacrylamide gel electrophoresis (SDS-PAGE) and transferred to polyvinylidene difluoride (PVDF) membranes (BioRad, Hercules, CA, USA). After blocking with 5% non-fat milk, the membranes were incubated overnight at 4 °C with the respective antibodies (Additional file 1: Table S2). Following incubation with an appropriate secondary antibody for 1 h at 25 °C, bound antibodies were detected using Pierce Western Blotting Substrate (Thermo Scientific).

### Plasmid construction and stable overexpression of HOXA5 short RNA

A cDNA library was prepared from HCT116 cells and the *HOXA5* short RNA was PCR amplified using the primer set including the forward primer 5′-AAAAACTCGAGGGGACCGGCGCCAGCTGCAGCCCGCCTCTTGCAGCCT-3′ (underline indicates XhoI site) and reverse primer 5′-AAAAAGGATCCGAACTTACAATAGAAAGTTTATTTTTTGTTCCAGTCAGTA-3′ (underline indicates BamHI site). The amplified products were cloned into the mammalian expression vector pEBMulti-Bsd. All constructs were confirmed by DNA sequencing. The plasmids were transfected into HCT116 cells using X-tremeGENE HP DNA Transfection Reagent (Roche Diagnostics) according to the manufacturer’s instructions. Stable transfectants were selected using blasticidin (5 μg/ml; InvivoGen, San Diego, CA, USA).

### Gene expression and pathway analyses

To specifically silence *HOXA5* short RNA, HCT116 cells were treated with siRNA #7, siRNA #8, or control siRNA for 48 h. Total RNA was extracted from the siRNAs-treated HCT116 cells and *HOXA5* short RNA stably overexpressed HCT116 cells (pEB-HOXA5 short or pEB-mock) as described above. After the quality of the purified RNA was assessed, gene expression was determined using a whole human genome microarray (SurePrint G3 Human; Agilent Technologies, Santa Clare, CA, USA) as described previously [15]. Microarray data were analyzed using GeneSpring 14.9 (Agilent Technologies). The mRNA signals within the lowest 20th percentile of all intensity values in at least half of the samples were excluded and the data set was filtered on the existing flag values. The microarray and sample annotation data have been deposited in Gene Expression Omnibus (GEO; accession number GSE124480). The functional pathways related to the set of differentially expressed genes were assessed by Ingenuity Pathway Analysis (IPA; https://www.qiagenbioinformatics.com/products/ingenuity-pathway-analysis/). The probability of a relationship between each biological function and the identified genes was calculated using Fisher’s exact tests. The level of statistical significance was set at a *P*-value ≤0.05.

### In vitro translation assay

In vitro translation was carried out using the FluoroTect Green in vitro Translation Labeling System (Promega) according to manufacturer’s instructions. The product contained a modified charged lysine tRNA labeled with the fluorophore BODIPY-FL. Using this system, fluorescently labeled lysine residues were incorporated into nascent proteins at multiple sites during translation. The products were separated by 15% SDS-PAGE and visualized using a Typhoon FLA 9500 laser scanner (GE Healthcare Life Sciences, Pittsburgh, PA, USA).

### Assessment of malignant phenotypes

Cell growth was assessed by counting the number of cells using a hemocytometer. Cell migration was examined using 8-μm pore size polycarbonate Transwell filters (Becton Dickinson, Franklin Lakes, NJ, USA). After serum starvation for 48 h, the cells were seeded in serum-free media onto the upper side of a Transwell chamber and allowed to migrate towards media containing 10% FBS in the lower chamber for 24 h. After migration, the cells on the lower side of the membrane were fixed, stained with Diff-Quick stain (Sysmex, Kobe, Japan), and counted. The migration indices were calculated as the mean number of cells in five random microscopic fields at 20 × magnification.

A xenograft mouse model was used to determine the tumor-forming capability of *HOXA5* short RNA-expressing cells. All procedures for the animal experiments were approved by the Animal Care Committee of the University of Tokushima. Seven-week-old male athymic nude mice (Nippon SLC, Shizuoka, Japan) were caged in groups of five and acclimated for one week. HCT116 cells stably expressing *HOXA5* short RNA (5 × 10^6^ cells in serum-free DMEM medium) were injected subcutaneously into the right or left flank of the nude mice (*n* = 5), and an equal number of cells stably expressing the mock construct was subcutaneously injected into the contralateral flank of the same mice. The sizes of developing tumors were measured in two dimensions using a caliper and their volumes were calculated as previously described [16]. After 30 days of injections, the mice were euthanized by CO2 asphyxiation to obtain tumor tissues. The fresh tissues were immediately frozen in liquid nitrogen and stored at − 80 °C until proteins were extracted for western blot analysis.

### Statistical analysis

Results were presented as mean ± standard deviations (SD). GraphPad Prism 6 (San Diego, CA, USA, USA) was used for all statistical analyses in this study. The differences in the means of RNAs expression, cell numbers, or tumor volumes were determined using the Student’s *t* test. *P* < 0.05 were considered statistically significant.

## Results

### Inhibition of cell growth in *HOXA5* knockdown cells

**Fig. 1:**
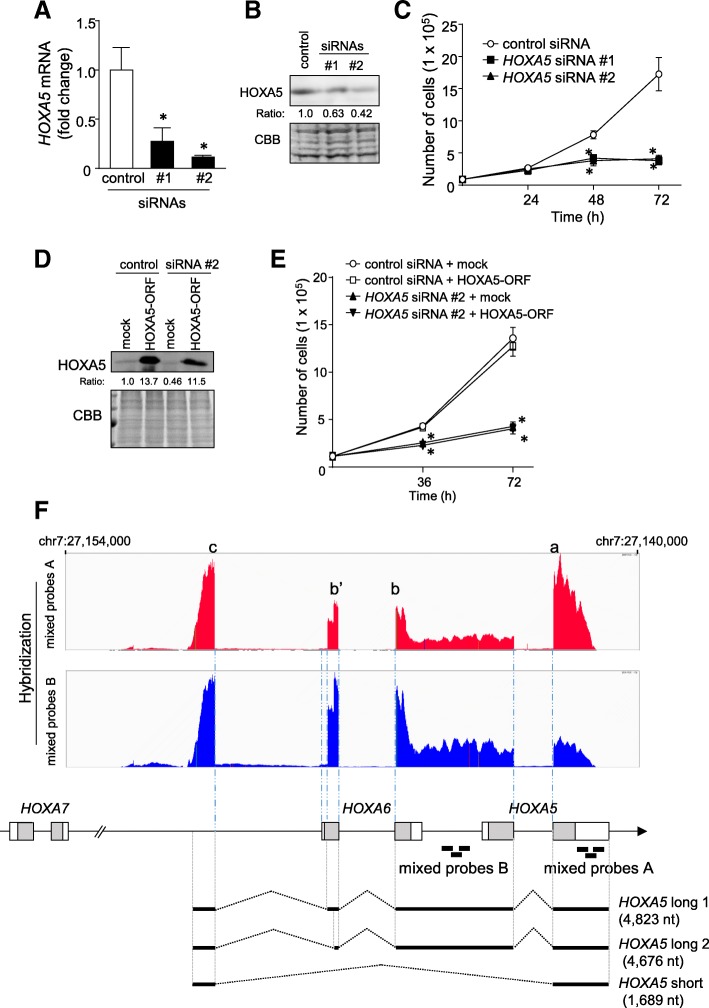
Analysis of *HOXA5* transcripts using a next generation sequencing-based targeted RNA-capture system. **a**. After treating HCT116 cells with the indicated siRNAs for 48 h, *HOXA5* mRNA levels were measured by qPCR, using *GAPDH* as an endogenous quantitative control. Data are expressed as the mean fold changes ± standard deviation (SD; *n* = 4), compared with those in control siRNA-treated cells. *Statistically significant difference versus control siRNA-treated cells (unpaired Student’s *t*-test, *P* < 0.01). **b**. The levels of HOXA5 protein were measured by western blotting. Coomassie brilliant blue (CBB) stain was used as a loading control. The amount of HOXA5 protein relative to that of CBB-stained bands was quantitatively analyzed by densitometry. **c**. After treating HCT116 cells with 10 nM of the indicated siRNAs, the cells were counted at the indicated times. Values are shown as the mean ± SD (n = 4). *Statistically significant difference versus control siRNA-treated cells (unpaired Student’s *t*-test, *P* < 0.01). **d** and **e**. HCT116 cells were treated following the rescue procedures described in the Methods section. HOXA5 protein levels in nuclear fractions were measured by western blotting (**d**). The cells were counted at the indicated times (**e**). The values shown represent the means ± SD (n = 4). *Statistically significantly difference versus control siRNA-treated and mock-transfected cells (unpaired Student’s *t*-test, *P* < 0.01). **f**. A DNA library was prepared using SMARTer Target RNA Capture with mixed probes **a** or **b**, as described in the Methods section. Sequence data from a MiSeq system were visualized using the Integrative Genomics Viewer. Sequence-read alignments from mixed probes A and B are indicated in red and blue, respectively. Grey boxes indicate HOXA5- and HOXA6-encoding sequences. Three transcripts (*HOXA5* long 1, long 2, and short) are represented under the genome schematic based on the Target RNA Capture analysis, 5′-RACE, and 3′-RACE assays used to define their sequences

HOXA5 controls proliferation and differentiation in a cell type-specific manner [4, 17]. After HCT116 cells were transfected for 72 h with two different siRNAs that targeted *HOXA5* exon 2 (#1) or 3′ UTR (#2), respectively, their growth rates were monitored. The two siRNAs reduced *HOXA5* mRNA expression and HOXA5 protein levels (Fig. 1a and b) and significantly inhibited cell growth (Fig. 1c).

To further confirm the growth inhibition following HOXA5 knockdown, we tested whether the overexpression of HOXA5 could rescue the growth inhibition in endogenous HOXA5 knockdown cells. Treatment with *HOXA5* siRNA #2 targeting the *HOXA5* 3′ UTR reduced HOXA5 protein levels and ectopic HOXA5 protein was sufficiently expressed (Fig. 1d). Unexpectedly, HOXA5 overexpression did not accelerate the growth of control siRNA-treated cells (Fig. 1e). Moreover, overexpressed HOXA5 failed to rescue the growth inhibition of endogenous HOXA5-silenced cells (Fig. 1e). These results suggested that *HOXA5* siRNA #1 and #2 inhibited the growth of HCT116 cells independent of HOXA5 protein.

### Identification of *HOXA5* transcripts

To explain the results described above, we searched for *HOXA5* transcript variant(s) containing the *HOXA5* 3′ UTR using a next generation sequencing based-RNA targeted capture system. Briefly, complementary oligonucleotide probes for the *HOXA5* 3′ UTR were designed (probes 1 to 3 in Fig. 1f and Additional file 1: Table S1). Total RNA was hybridized with the target-specific biotinylated DNA probes (mixed probes A) and then purified prior to capture with magnetic beads coated with streptavidin. A cDNA library was prepared from the captured RNA samples and subjected to next-generation sequencing analysis. The obtained reads were aligned and mapped to the human genome (hg38) using IVG software. As shown in Fig. 1f, two major peaks were detected corresponding to *HOXA5* exon 2 (peak a in Fig. 1f) and the upstream region of *HOXA6* (peak c), which suggested the presence of a short splice variant that skipped *HOXA5* exon 1. Minor peaks corresponding to *HOXA6* exon 1 and 2 (peaks b and b′) were also detected. Additionally, analysis of the next-generation sequencing reads detected significant number of transcripts spanning the entire intergenic region between *HOXA5* and *HOXA6* (between peaks a and b). These results suggested the presence of long transcript(s) containing *HOXA5*, *HOXA6*, and the entire intergenic region between the two genes. To validate the presence of transcripts containing the upstream region of *HOXA5*, we again employed RNA targeted capture sequencing with probes targeting regions upstream of *HOXA5* (mixed probes B in Fig. 1f and Additional file 1: Table S1). As shown in Fig. 1F, an enriched peak (corresponding to peak c) was again observed using the mixed probes B, which targeted the region upstream of *HOXA5*, suggesting that transcripts containing sequences upstream of *HOXA5* may be transcribed from the region upstream of *HOXA6*.

To validate the predicted novel transcripts, we used 5′-RACE and 3′-RACE techniques. First, we performed 5′-RACE using primers specific for *HOXA5* exon 1 (5′-3), *HOXA5* exon 2 (5′-2), and the upstream region of *HOXA5* (5′-1) as detailed in Additional file 2: Figure S2A. A single transcript was amplified, as indicated by the arrow “a” in Additional file 2: Figure S2B, when the 5′-1 primer was used. The 5′-RACE with primer 5′-3 also detected a single PCR product (arrow “b” in Additional file 2: Figure S2B). Sanger sequence analysis revealed that the band “a” product included two different splice variants of *HOXA6* exon 1 (long 1 and long 2). Sanger sequencing also identified *HOXA5* short RNA (short), in which exon 1 of *HOXA5* was skipped (Additional file 2: Figure S2C). All three transcripts commonly used a transcriptional start site located in the intergenic region between *HOXA7* and *HOXA6* (Fig. 1f and Additional file 2: Figure S2C). However, no transcripts were detected when the 5′-2 primer was used.

We then performed 3′-RACE experiments using primers 3′-1 and 3′-2 to determine the 3′-terminus for each of the novel transcripts (Additional file 2: Figure S3A). Although 3′-RACE experiments in which the 3′-1 primer was used resulted in the amplification of “c” bands that consisted of two different lengths, cloning and sequencing identified a single transcript (Additional file 2: Figure S3C). The band “d” corresponded to an exon 1-skipped *HOXA5* short RNA (Additional file 2: Fig. S3B and C). These transcripts use the same annotated polyA site of the *HOXA5* gene (Fig. 1f and Additional file 2: Figure S3).

Based on the results of the 5′-RACE and 3′-RACE experiments, we developed a schematic of the *HOXA5* transcripts detected in this series (Fig. 1f). The transcripts included a 1689-nt transcript (*HOXA5* short), a 4823-nt transcript overlapping *HOXA6* and *HOXA5* (*HOXA5* long 1), and a 4676-nt transcript lacking a portion of *HOXA6* exon 1 (*HOXA5* long 2).

### Role of *HOXA5* transcripts in cell growth

**Fig. 2:**
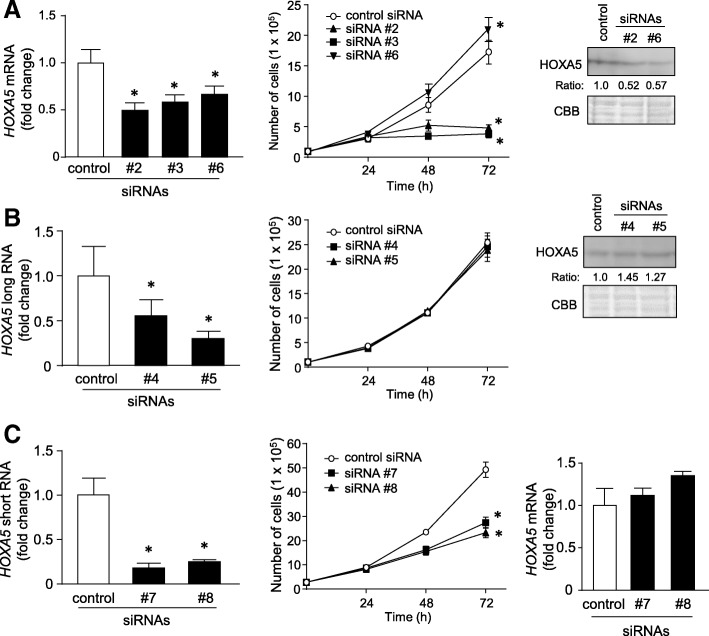
Knockdown of *HOXA5* short RNA inhibited cell growth. **a** and **b** (left panels) and **c** (left and right panels). After HCT116 cells were treated with 10 nM of the indicated siRNAs for 48 h, expression levels of *HOXA5* mRNA, *HOXA5* long RNA, and *HOXA5* short RNA were measured by qPCR using *GAPDH* mRNA as an endogenous quantitative control. Data are expressed as the mean fold changes ± standard deviation (SD; *n* = 4) compared with those in the control siRNA-treated cells. *Statistically significantly difference versus control siRNA-treated (unpaired Student’s *t*-test, *P* < 0.05). **a**, **b**, and **c** (middle panels). HCT116 cells (1.0 × 10^5^ cells) were seeded into 35-mm-diameter dishes and transfected with 10 nM of the indicated siRNAs or control siRNA. Subsequently, the growing cells were counted at the indicated times. Values are means ± SD (*n* = 4). *Statistically significantly difference versus control siRNA-treated (unpaired Student’s *t*-test, *P* < 0.01). **a** and **b** (right panels). After HCT116 cells were treated with 10 nM of the indicated siRNAs for 48 h, nuclear fractions were prepared from the cells. The levels of HOXA5 protein were measured by western blotting. The same amounts of protein used for western blotting were subjected to SDS-PAGE followed by CBB staining, for a loading control. The amount of HOXA5 protein relative to that of CBB stained bands was quantitatively analyzed by densitometry

Both the next generation sequencing based-RNA targeted capture system and the 5′-RACE and 3′-RACE analyses failed to detect a *HOXA5* mRNA that was expected to be transcribed from the 5′-flank of *HOXA5* (Ensembl ID: ENST00000222726). HOXA5 expression is reported to be suppressed in poorly differentiated colon cancer cells, such as HCT116 cells [9]. Here, HCT116 cells expressed only a small amount of HOXA5 protein (right panels of Fig. 2a and b). Therefore, we focused on the function of the newly identified transcripts using siRNAs (Additional file 2: Figure S1). Specifically, siRNA #2 and #3 targeted common sequences of all three *HOXA5* transcripts, siRNA #6 targeted *HOXA5* exon 1, and siRNA #4 and #5 targeted *HOXA5* long 1 and long 2. To silence the *HOXA5* short RNA, siRNA #7 and #8 targeted the junction sequences between the first and last exon. Knockdown efficiency of each siRNA was monitored by measuring mRNA levels of each *HOXA5* transcript using qPCR (Fig. 2a, b, and c, left panels).

Knockdown of all *HOXA5* transcripts with siRNA #2 and #3 significantly inhibited cell growth (*P* < 0.01)*.* As shown in the right panel of Fig. 2a, treatment with siRNA #2 or #6 reduced HOXA5 protein levels. Contrastingly, treatment with siRNA #6, which also silenced *HOXA5* mRNA, accelerated cell growth (Fig. 2a). The reduction in levels of the long isoforms (*HOXA5* long 1 and long 2) by treatment with siRNA #4 and #5 did not affect cell growth or HOXA5 protein levels (Fig. 2b). It should be noted that selective knockdown of the short isoform (*HOXA5* short) with either siRNA #7 or #8 significantly inhibited cell growth without affecting *HOXA5* mRNA levels (*P* < 0.01; Fig. 2c). These complex findings from the experiments suggested the following possibilities. First, although HCT116 cells expressed lower levels of HOXA5 protein, the reduction of HOXA5 accelerated cell growth, consistent with that reported by Teo et al. [18]. Second, *HOXA5* long 1 and long 2 RNAs were not translated into HOXA5 protein. Third, *HOXA5* short RNA played an important role in the regulation of HCT116 cell growth, independent of HOXA5 protein. Based these findings, we focused on the function of *HOXA5* short RNA in subsequent experiments.

### The *HOXA5* short RNA was untranslatable

To examine whether the *HOXA5* short RNA was translated as a truncated HOXA5 protein, we conducted in vitro transcription/translation assays. In brief, amplified PCR products of the full-length of *HOXA5* short RNA sequences were incubated with rabbit reticulocyte lysate (Promega). Fluoro-labeled lysine was added to allow for visualization of the synthesized proteins. As shown in Additional file 2: Figure S4A, no detectable protein product was translated from the *HOXA5* short RNA template.

We also investigated the potential of the *HOXA5* short RNA to be translated into protein using the web-based software, Coding-Potential Assessment Tool (CPAT). As shown in Additional file 2: Figure S4B, CPAT predicted that *HOXA5* short RNA had a very low coding potential, which was similar to *MALAT1*, a well-known lncRNA. These data suggested that the *HOXA5* short transcript may play a role in cell proliferation as a functional lncRNA.

### Expression of *HOXA5* short RNA enhanced cell proliferation and cell migration

**Fig. 3:**
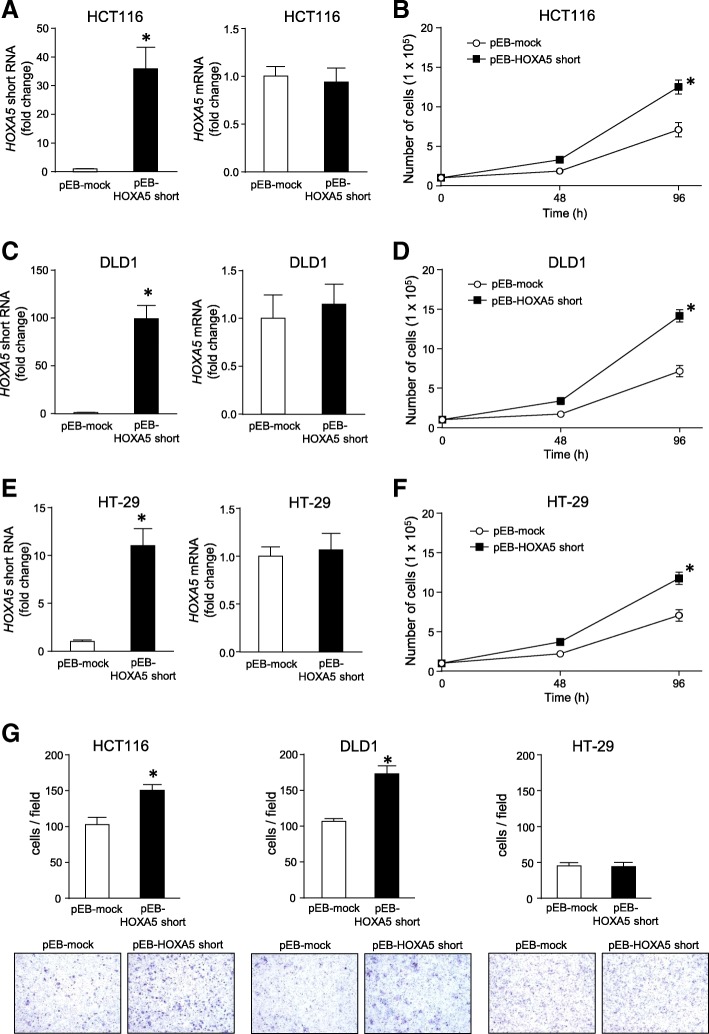
Regulation of cell proliferation and migration by stably overexpressed *HOXA5* short RNA. After HCT116 (**a**), DLD1 (**c**), and HT-29 (**e**) cells were transfected with pEB-HOXA5 short or pEB-mock vector and selected using blasticidin, expression levels of *HOXA5* short RNA and *HOXA5* mRNA were measured by qPCR using *GAPDH* mRNA as an endogenous quantitative control. Data are expressed as the mean fold changes ± standard deviation (SD; *n* = 4) compared with those in the pEB-mock cells. *Statistically significantly difference versus the pEB-mock cells (unpaired Student’s *t*-test, *P* < 0.01). **b**, **d**, and **f**. The indicated cells (1.0 × 10^5^ cells) were seeded into 35-mm-diameter dishes and the growing cells were counted at the indicated times. Values are means ± SD (*n* = 4). *Statistically significantly difference versus the pEB-mock cells (unpaired Student’s *t*-test, *P* < 0.01). **g**. After the indicated cells were cultured with serum-free medium for 36 h, they were seeded in serum-free medium onto the upper side of a Transwell chamber and allowed to migrate towards 10% FBS-containing medium in the lower chamber. After incubation for 24 h, the migrating cells were stained with Diff-Quick dye (lower panels) and counted (upper panel). Data are presented as the means ± SD (*n* = 4). *P < 0.05, unpaired Student’s *t*-test

To confirm the mitotic potential of the *HOXA5* short RNA, we established three different colon cancer cell lines that stably expressed the *HOXA5* short RNA transcript using HCT116, DLD1, and HT-29 cells. Compared to that in the mock-transfected cells, *HOXA5* short RNA levels were increased in all three established cell lines without changing the levels of coding *HOXA5* mRNA (Fig. 3a, c, and e). Overexpression of *HOXA5* short RNA significantly facilitated the growth of HCT116, DLD1, and HT-29 cells (*P* < 0.01; Fig. 3b, d, and f, respectively). We also examined whether increased expression of *HOXA5* short RNA increased the migration capability of cells using the Boyden chamber method and found that overexpression of *HOXA5* short RNA increased the migration of HCT116 and DLD1 cells, but not HT-29 cells (Fig. 3g).

### *HOXA5* short RNA was a potential activator of EGF signaling

**Fig. 4:**
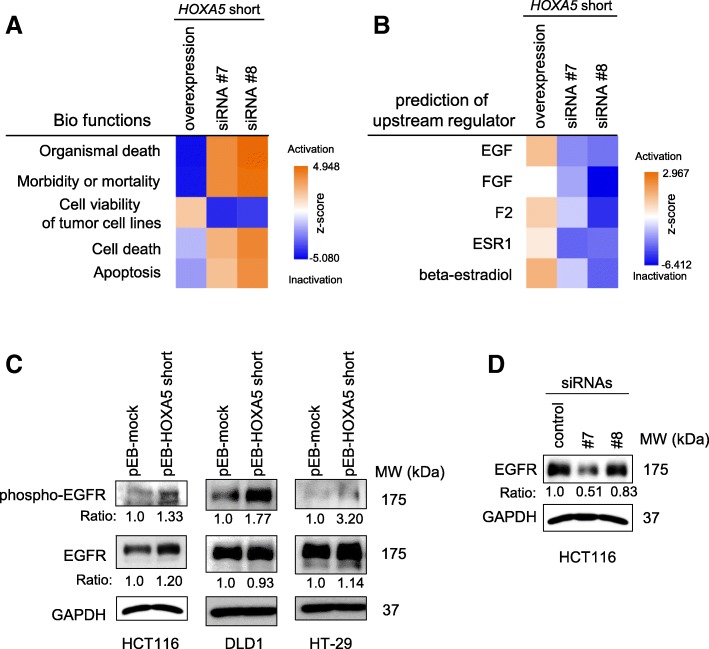
*HOXA5* short RNA activated epidermal growth factor (EGF) signaling. **a** and **b**. Gene expression profiles in *HOXA5* short RNA-overexpressing cells and silencing cells (siRNA #7 and siRNA #8) were analyzed using a whole human genome microarray (Agilent Technologies) and GeneSpring 14.9 (Agilent Technologies). IPA analysis of the top-five ranked bio-functions (**a**) and predicted upstream regulators (**b**) for the differentially expressed genes between the two types of cells. **c** and **d**. Protein levels of phosphorylated EGF receptor (EFGR) and total EGFR were measured by western blot analysis. GAPDH levels were used as an endogenous quantitative control. The level of phospho-EGFR or EGFR band relative to that of GAPDH was quantitatively analyzed by densitometry as indicated

To elucidate the mechanism of *HOXA5* short RNA-induced acceleration of cell growth and migration, we analyzed differences in gene-expression profiles between siRNA #7-treated or #8-treated HCT116 cells (*HOXA5* short RNA-silenced) and HCT116 cells stably expressing *HOXA5* short RNA. IPA analysis of differentially expressed genes indicated that *HOXA5* short RNA activated canonical pathways of “Cell viability of tumor cell lines” and inhibited those of “Organismal death”, “Morbidity and mortality”, “Cell death”, and “Apoptosis” (Fig. 4a). These results were consistent with the observed changes in phenotypes following the reduction or overexpression of the *HOXA5* short RNA. Furthermore, as shown in Fig. 4b, IPA identified EGF as an upstream regulator when z-scores were considered (2.484 in *HOXA5* short RNA-overexpressing cells, − 2.907 in siRNA #7-treated cells, and − 3.53 in siRNA #8-treated cells). IPA suggested that *EGFR* mRNA expression was upregulated in *HOXA5* short RNA-overexpressing HCT116 cells compared to that in the mock-transfected cells (log2 fold-change = 1.121). It is well known that EGFR plays a crucial role in epithelial malignancies through facilitating cell proliferation and invasion [19]. Therefore, we assessed protein levels of EGFR in *HOXA5* short RNA-expressing HCT116, DLD1, and HT-29 cells. The expression of *HOXA5* short RNA enhanced phosphorylation of EGFR in the HCT116, DLD1, and HT-29 cells. EGFR protein levels were increased in the HCT116 and HT-29 cells, but not in the DLD1 cells, when *HOXA5* short RNA was overexpressed. (Fig. 4c). In contrast, silencing *HOXA5* short RNA reduced EGFR levels (Fig. 4d). These observations suggested that *HOXA5* short RNA may have promoted cell proliferation and migration through stimulation of the EGF signaling pathway.

### Enhanced expression of *HOXA5* short RNA in cancer cells and advanced colon tissues

**Fig. 5:**
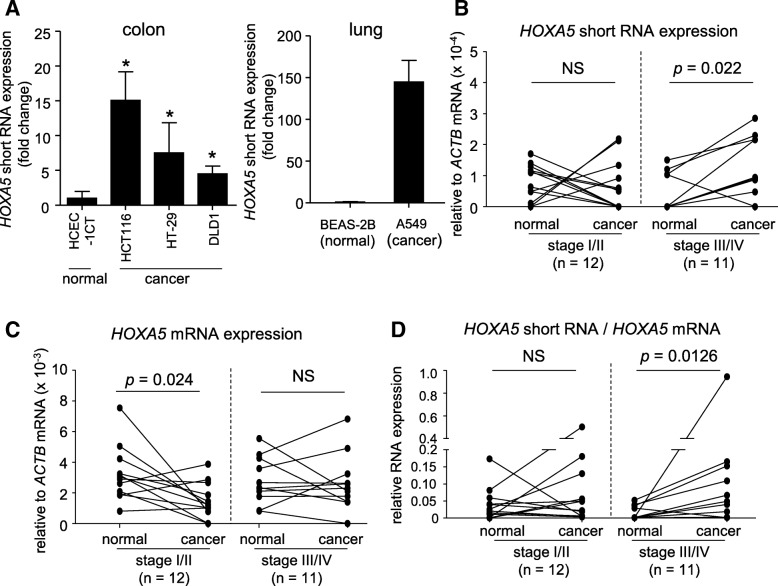
Expression of *HOXA5* short RNA in cancer cell lines and colon cancer tissues. **a**. *HOXA5* short RNA expression in normal colonic epithelial (HCEC-1CT), colorectal cancer cells (HCT116, DLD1 and HT-29), normal bronchial epithelial (BEAS-2B), and lung cancer (A549) cells was measured by qPCR using *GAPDH* mRNA as an endogenous quantitative control. Data are expressed as the mean fold changes ± standard deviation (SD; *n* = 4) compared with those in the HCEC-1CT cells or BEAS-2B. (*P < 0.05, Dunnett’s test (colon) or unpaired Student’s *t*-test (lung)). **b**, **c**, and **d**. The expression of *HOXA5* short RNA (**b**) and *HOXA5* mRNA (**c**) in cDNA libraries prepared from colon cancers and surrounding normal colonic mucosa from 23 patients were measured by qPCR. *ACTB* mRNA was used as an endogenous quantitative control. Relative expression of *HOXA5* short RNA to *HOXA5* mRNA are shown in **d**. *P values were calculated by the paired Student’s *t*-test. NS = not statistically significant difference

When *HOXA5* short RNA levels were compared among colorectal cancer cells (HCT116, DLD1 and HT-29) and normal colonic epithelial cells (HCEC-1CT), it was determined that cancer cells expressed higher levels of *HOXA5* short RNA than normal cells (Fig. 5a). A549 lung cancer cells also showed high expression of *HOXA5* short RNA when compared with normal lung epithelial cells (BEAS-2B) (Fig. 5a). To further investigate the potential clinical relevance of *HOXA5* short RNA in tumor development, we measured the expression of both *HOXA5* short RNA and coding *HOXA5* mRNA in cDNA libraries prepared from 21 patients with colon cancer (HCRT103; OriGene, Rockville, MD, USA). As shown in Fig. 5b, advanced colon cancer tissues (stage III or IV) expressed significantly higher levels of *HOXA5* short RNA compared to that in the paired normal tissues (*P* = 0.022), whereas the expression of the coding *HOXA5* mRNA was downregulated during early stages (*P* = 0.024) and remained unchanged in colon cancer tissues from advanced stages (Fig. 5c). Additionally, the expression of *HOXA5* short RNA relative to *HOXA5* mRNA was increased in advanced colon cancer tissues (*P* = 0.0126; Fig. 5d). These data suggested that high expression of *HOXA5* short RNA may be involved in tumor growth.

### *HOXA5* short RNA enhanced tumor growth in vivo

**Fig. 6:**
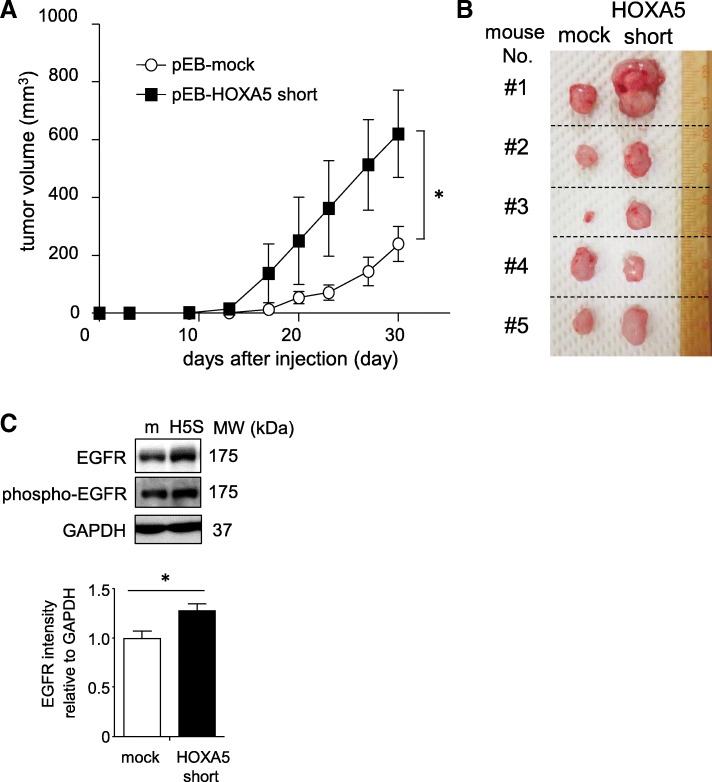
*HOXA5* short RNA enhanced tumor growth in vivo. **a**. Tumor growth was assessed up to 30 d post inoculation and the tumor volume (mm^3^) was calculated. Changes in tumor masses over time are shown in **a**. Values are means ± standard error of the mean (SEM; *n* = 5). Growing tumors from five mice on day 30 post inoculation are presented in **b** (*P < 0.05, unpaired Student’s *t*-test). **c**. Tissue lysates was prepared and subjected to western blot analysis. Protein levels of the phosphorylated EGFR and total EGFR were analyzed by densitometry. Results are shown in the bar graph in panel **c** (*P < 0.05, unpaired Student’s *t*-test). m, mock; H5S, HOXA5 short

To demonstrate the function of *HOXA5* short RNA on tumor development in vivo, male athymic nude mice (*n* = 5) were subcutaneously injected with HCT116 cells stably expressing *HOXA5* short RNA (pEB-HOXA5 short) and mock-transfected HCT116 (pEB-mock) cells and the growing tumor masses were measured. The pEB-HOXA5 short cells rapidly and progressively developed into tumors. The average volumes of the pEB-HOXA5 short cell tumors were significantly larger than those of the pEB-mock cell tumors 30 d post grafting (*P* < 0.05) (Fig. 6a and b). As shown in Fig. 6c, the stable overexpression of *HOXA5* short RNA increased EGFR levels and facilitated EGFR phosphorylation in the xenograft tumors.

## Discussion

HOXA5 plays a crucial role in the regional specification and organogenesis during embryo development [20, 21]. Additionally, dysregulated HOXA5 expression has been observed in several types of cancers and is associated with their progression. Here, we identified a novel transcript derived from the *HOXA6*-*HOXA5* locus using a next generation sequencing-based targeted RNA capture method and determined its function in cell proliferation and migration in vitro and in vivo*.* A previous study has shown that *HOXA5*^−/−^ mice do not exhibit accelerated rates of spontaneous tumorigenesis [22], suggesting that the loss of HOXA5 function may not be sufficient to initiate carcinogenesis. However, HOXA5 expression has been shown to be progressively downregulated during the adenoma-carcinoma transition in colon tissue [9], suggesting that HOXA5 protein may function as a tumor suppressor protein. Here, treatment of cells with siRNAs that targeted *HOXA5* exon 2 and its 3′ UTR inhibited cell growth; however, this inhibitory effect could not be rescued by the ectopic expression of HOXA5 protein. Thus, we speculated that HOXA5 protein levels may not be involved in the HOXA5 siRNA-induced inhibition of cell growth.

Coulombe et al. have revealed that complex transcriptional units encompassing the *Hoxa6*-*Hoxa5* locus exist in the mouse embryo [12]. They have identified a distal promoter (D2) located upstream of *Hoxa6*, which is highly conserved among the species. However, we determined that the transcriptional start site of the *HOXA5* short RNA was located downstream of the D2 promoter. This *HOXA5* short RNA has not been previously documented. A putative TATA box resides at position − 54 to − 39 relative to the transcriptional start site of the *HOXA5* short RNA. The nucleotide sequences around the TATA box are highly conserved among mammals (Additional file 2: Figure S5). The *HOXA5* short RNA was not translated into protein in the in vitro transcription/translation assay. The CPAT index supported this result (Additional file 2: Figure S4B). Thus, the 1648-nt *HOXA5* short RNA was likely to be a functional lncRNA.

Recently, lncRNAs have been recognized as critical players in cancer development through the regulation of transcription, RNA processing, translation, and chromatin modification [23]. Interestingly, Xu et al. reported that 48 *HOX*-related non-coding RNAs (ncRNAs) are aberrantly expressed in lung adenocarcinoma [24]. These ncRNAs may be able to disturb the fine tuning of *HOX* cluster gene expression. Rinn et al. have shown that the lncRNA *HOTAIR*, which is transcribed from the *HOXC* cluster, regulates *HOXD* gene expression *in trans* [25]. In our experiment, gene expression analysis using microarray showed that *HOXA13*, *HOXB7,* and *HOXD12* were downregulated, and *HOXA6*, *HOXB4,* and *HOXB9* were upregulated in *HOXA5* short RNA knockdown cells. It is possible that *HOXA5* short RNA may modify expression of these *HOX* genes.

*HOXA5* short RNA was highly expressed in colon cancer cells and advanced colon cancer tissues (Fig. 5), suggesting that *HOXA5* short RNA plays an oncogenic role. To further determine the pathophysiological significance of *HOXA5* short RNA, we also assessed resistance to 5-fluorouracil (5-FU) in wild-type and stably transfected HCT116 cells; however, overexpression of *HOXA5* short RNA failed to change the level of 5-FU resistance (Additional file 2: Figure S6).

Analysis of differentially expressed genes in HCT116 cells overexpressing *HOXA5* short RNA compared to cells in which *HOXA5* short RNA was silenced revealed that the expression of EGF signal-related genes was prominently different between the two types of cells. EGFR plays crucial roles in epithelial malignancies, including tumor growth, invasion, and metastasis, through stimulation of downstream signaling cascades, such as ERK or AKT pathways [19, 26, 27]. However, under the current experimental conditions, *HOXA5* short RNA dramatically altered neither ERK nor AKT activation (Additional file 2: Figure S7). IPA showed no activation of ERK or AKT signaling at the mRNA level. Further studies are needed to reveal the mechanistic consequence of EGFR phosphorylation promoted by *HOXA5* short RNA. IPA also predicted the upregulation of both ESR1 (estradiol receptor alpha) and estradiol. ESR1 increases cellular proliferation in tumor cell lines, such as lung [28], prostate [29] and breast [30] cancer cells. However, ESR1 function in colon cancer has not been elucidated, because its expression was limited in normal and malignant colonic epithelium [31]. Several lncRNAs have been recently identified, and distinct lncRNAs play crucial roles in various biological functions and diseases, including cancer [23]. However, their functions are not fully understood. The regulation of gene expression by lncRNAs occurs through various mechanisms. One emerging theme is that lncRNAs modify cell signaling pathways through the formation of RNA-protein complexes or through the modification of protein-protein interactions. For instance, Lin et al. have suggested that long intergenic ncRNA for kinase activation *(LINK-A*) can activate AKT by facilitating direct interaction between the AKT pleckstrin homology domain and phosphatidylinositol (3,4,5)-trisphosphate [32]. However, currently lncRNA-mediated regulation of EGF signaling has not been documented.

In addition to *HOXA5* short RNA, we also found two long isoforms that were transcribed from the *HOXA6*-*HOXA5* locus, which we referred to as *HOXA5* long 1 and *HOXA5* long 2. Coulombe et al. showed that among several isoforms of *HOXA5* that have been reported in the mouse embryo, only the isoform transcribed just upstream of exon 1 of *HOXA5* is translated into protein when transfected into HEK293 cells [12]. This translatable isoform corresponds to Hoxa5–201 (Transcript ID: ENSMUST00000048794) in mice and to HOXA5–201 (ENST00000222726) in humans. However, 5′-RACE experiment failed to detect the transcriptional start site of HOXA5–201 under our experimental conditions. Treatment of cells with *HOXA5* siRNA #2, which targeted *HOXA5* 3′UTR, and siRNA #6, which targeted *HOXA5* exon 1, resulted in reduced protein levels of HOXA5 (Fig. 2a). Contrastingly, treatment of cells with *HOXA5* siRNA #4 or #5, which targeted *HOXA5* long RNAs, did not decrease HOXA5 protein levels (Fig. 2b). These observations suggested that *HOXA5* long RNAs may be untranslatable transcripts. Additional studies are needed to further characterize these long RNAs.

## Conclusions

We found novel transcripts from *HOXA5* in human colon cancer HCT116 cells using a next generation sequencing-based targeted RNA capture system. Our results indicated that a novel transcript named *HOXA5* short RNA could regulate cell proliferation as a functional lncRNA both in vitro and in vivo. Furthermore, we provide evidence that *HOXA5* short RNA may have activated EGFR signaling in both colon cancer cell lines and xenograft tumors. To our knowledge, this is the first study to determine the full-length sequence of the *HOXA5* short RNA and to reveal its function as an oncogenic lncRNA. Although further studies are needed to fully define the role of *HOXA5* short RNA in the process of colon cancer, this study provides new insight into the potential role of *HOXA5*-derived functional RNAs in colon cancer growth.

## Additional files

Additional file 1: **Table S1.** Primer sets used for qPCR, primers for 5′- and 3′-RACE, and oligonucleotide sequences for siRNAs and biotinylated DNA probes. **Table S2.** List of primary antibodies used in Western Blotting analysis. (DOC 69 kb)

Additional file 2: **Figure S1.** Schematic representation of the primer sets used for the qPCR assay and siRNAs targeting of the *HOXA6-HOXA5* locus. Primers for qPCR amplification of the indicated transcripts or genomic regions are indicated by the left/right arrows. Transcripts, *HOXA5* short and *HOXA5* long 1; regions, *HOXA5* coding region (CR) and 3′ UTR. Targeted sequences of siRNAs #1 to #8 are indicated by the solid lines and the dashed lines for siRNAs #7 and #8 indicate the skipped sequences of the siRNAs. **Figure S2.** 5′-rapid amplification of cDNA ends (RACE) experiments of the *HOXA6-HOXA5* locus. **A.** Scheme diagram of the gene-specific primers used for 5**′**-RACE experiment. **B.** Electrophoretic analysis of PCR amplification products. **C.** Nucleotide sequences of the PCR products. Primers used are underlined. Grey boxes indicate the junctions between different exons. M, DNA ladder marker. **Figure S3.** 3′-rapid amplification of cDNA ends (RACE) experiments of the *HOXA6-HOXA5* locus. **A.** Scheme diagram of the gene-specific primers used for 3**′**-RACE experiment. **B.** Electrophoretic analysis of PCR amplification products. **C.** Nucleotide sequences of the PCR products. Primers used are underlined. Grey boxes indicate the junctions between different exons. M, DNA ladder marker. **Figure S4.** Analysis of translation potency of the *HOXA5* short RNA. **A.** A T7 promoter-containing DNA fragments encoding full-length HOXA5 RNA, *HOXA5* short RNA, or GAPDH were generated by PCR amplification and the resultant PCR products were subjected to in vitro transcription and translation assays, which included the incorporation of fluorescent lysine. The synthesized proteins were analyzed by 15% SDS-PAGE and detected using a fluoro-imaging instrument. **B.** The translation potency of *HOXA5* short RNA was calculated using Coding-Potential Assessment Tool (CPAT) software. Sequences of the coding regions of *HOXA5* and *GAPDH* were used as translatable sequences and that of *MALAT1,* known as a functional long non-coding RNA, was used as an untranslatable sequence. **Figure S5.** Evolutionary conserved sequences of a transcriptional start site of the *HOXA5* short RNA. Sequence alignment of the upstream sequences of a transcriptional start site (TSS) in *HOXA5* short RNA indicates the presence of a consensus TATA box and a TSS in most species. **Figure S6.** Intrinsic chemoresistance to 5-FU in HOXA5 short RNA expressing HCT116 cells. The cell viability of pEB-HOXA5 short or pEB-mock HCT116 cells was determined by Cell Count Reagent SF after treatment with increasing doses of 5-FU for 48 h. **Figure S7.** Effects of *HOXA5* short RNA on AKT and ERK activation. Protein levels of phosphorylated AKT (Ser473; #9271, Cell Signaling Tech.), total AKT (#9272, Cell Signaling Tech.), phosphorylated ERK1/2 (#9101, Cell Signaling Tech.) and total ERK1/2 (#9102, Cell Signaling Tech.) were measured by western blot analysis. GAPDH levels were used as an endogenous quantitative control. The level of phospho-AKT, phosphor-ERK1/2, AKT or ERK1/2 band relative to that of GAPDH was quantitatively analyzed by densitometry. #: The band corresponding to phospho-AKT was not sufficiently detected for densitometry analyses. (PDF 561 kb)

## Abbreviations

5-FU: 5-fluorouracil
cDNA: complementary DNA
CPAT: Coding-Potential Assessment Tool
DMEM: Dulbecco’s Modified Eagle Medium
EFGR: epidermal growth factor receptor
EGF: epidermal growth factor
FBS: fetal bovine serum
GAPDH: glyceraldehyde 3-phosphate dehydrogenase
gDNA: genomic DNA
GEO: Gene Expression Omnibus
HOXA5: Homeobox A5
IGV: Integrative Genomics Viewer
IPA: Ingenuity Pathway Analysis
lncRNA: long non-coding RNA
NGS: next-generation sequencing
PCR: polymerase chain reaction
PVDF: polyvinylidene difluoride
qPCR: quantitative real-time reverse transcription-PCR
RACE: rapid amplification of cDNA ends
SD: standard deviation
SDS-PAGE: sodium dodecyl sulfate–polyacrylamide gel electrophoresis
SEM: standard error of the mean
siRNAs: small interfering RNAs
UTR: untranslated region
