# Supplementary material for: A novel long non-coding RNA from the HOXA6-HOXA5 locus facilitates colon cancer cell growth
Source: BMC Cancer. 2019 Jun 3;19:532. doi: 10.1186/s12885-019-5715-0 (PMC6547586; doi:10.1186/s12885-019-5715-0)
Supplement: Supplementary file 1 — Table S1. Primer sets used for qPCR, primers for 5′- and 3′-RACE, and oligonucleotide sequences for siRNAs and biotinylated DNA probes. Table S2. List of primary antibodies used in Western Blotting analysis. (DOC 69 kb) [file 12885_2019_5715_MOESM1_ESM.doc]

**Additional file 1: Table S1. Primer sets used for qPCR, primers for 5’- and 3’-RACE, and oligonucleotide sequences for siRNAs and biotinylated DNA probes**

|  |  | |  | |  |  |
| --- | --- | --- | --- | --- | --- | --- |
|  | Primers for qPCR | | | |  |  |
|  | Targets | |  | | primer sequences (5' - 3') |  |
|  | *HOXA5* 3’UTR | | forward | | TGAAGAAGCCCTGTTCTCGT |  |
|  |  | | reverse | | TTGAAGGGGGACTTTTTGTG |  |
|  | *HOXA5*_CR | | forward | | CCCAACCCCAGATCTACCC |  |
|  |  | | reverse | | GGGTCAGGTAACGGTTGAAG |  |
|  | *HOXA5_long* | | forward | | CAGGCTGGATACGTAAGCAAA |  |
|  |  | | reverse | | CCTGCCGCTCTTAAATGACA |  |
|  | *HOXA5_short* | | forward | | GAGGTGGAGCTGTTCAATGG |  |
|  |  | | reverse | | GGGTCAGGTAACGGTTGAAG |  |
|  | *GAPDH* | | forward | | AGCCACATCGCTCAGACAC |  |
|  |  | | reverse | | GCCCAATACGACCAAATCC |  |
|  |  | |  | |  |  |
|  | Gene-specific primers for 5’ RACE | | | | |  |
|  | Name |  | | | primer sequences (5' - 3') |  |
|  | 5’-1 | reverse | | | GGGTTACTGGGGTCTTGCTT |  |
|  | 5’-2 | reverse | | | TCGCTCACGGAACTATGATCT |  |
|  | 5’-3 | reverse | | | GGGTCAGGTAACGGTTGAAG |  |
|  |  |  | | |  |  |
|  | Gene-specific primers for 3’ RACE | | | | |  |
|  | Name | |  | | primer sequences (5' - 3') |  |
|  | 3’-1 | | forward | | GACTCCACTTGCCTCTCAGC |  |
|  | 3’-2 | | forward | | GAGGTGGAGCTGTTCAATGG |  |
|  |  | | | |  |  |
|  | Sequence of siRNAs | | | |  |  |
|  | Name | | |  | Sequence (5'-3') |  |
|  | *HOXA5* siRNA #1 | | |  | ACCCGCAGAAGGAGGAUUGAA |  |
|  | *HOXA5* siRNA #2 | | |  | UCCCGCGUAGUGUCAGUACUA |  |
|  | *HOXA5* siRNA #3 | | |  | AGGAGGAUUGGCUAUGGCAAA |  |
|  | *HOXA5* siRNA #4 | | |  | GGGCAAAGCACUCCAUGACGA |  |
|  | *HOXA5* siRNA #5 | | |  | AAACCAUAUUCACACGAAA |  |
|  | *HOXA5* siRNA #6 | | |  | UACGGCUACAAUGGCAUGGAU |  |
|  | *HOXA5* siRNA #7 | | |  | GAGAAACUGAAAGACAACAUA |  |
|  | *HOXA5* siRNA #8 | | |  | AACUGAAAGACAACAUAGGCG |  |
|  |  | | |  |  |  |
|  | Sequence of biotinylated DNA probes for target RNA capture | | | | |  |
|  | Name |  | sequence (5’-3’) (5’ end labeled by a biotin) | | |  |
|  | probe 1 |  | GCTTGGAGCTATTGAGACAGGAACACTTCCACGCACATGCACAGTTAAACAACTTGAGTG | | |  |
|  | probe 2 |  | CAGGGACACACCGCTTGGAGTCACAGTTTTCATCACAGAGTCACTAGTCACTACACGTCG | | |  |
|  | probe 3 |  | TGAAGCTGCGCTTATAAGAGCCACTTCCAGAGTTCGTGCAAAGGGTCCTATAAAGGCACG | | |  |
|  | probe 4 |  | GTGTGAATATGGTTTTCGTGTCATTAGTTTGCGATTTGATTTGCTTACGTATCCAGCCTG | | |  |
|  | probe 5 |  | TCCAAGGCGAGGTCAAATTCCATACACTTTTATAACCGTAGTCGATTTTTCTTTCGTGTG | | |  |
|  | probe 6 |  | CTGCCGCTCTTAAATGACAGGCGTCTATTAAAGATAGCTTTTGTGTAGTGTTTCTCCAAG | | |  |

Table S2: List of primary antibodies used in Western Blotting analysis.

|  |  |  |  |  |
| --- | --- | --- | --- | --- |
|  | Target | Dilution | Commercial supplier |  |
|  | HOXA5 | 1 : 500 | sc-365784, Santa Cruz Biotechnology |  |
|  | EGFR | 1 : 1000 | #4267, Cell Signaling Technology |  |
|  | phospho-EGFR (Tyr1068) | 1 : 500 | #3777, Cell Signaling Technology |  |
|  | GAPDH | 1 : 5000 | sc-47724, Santa Cruz Biotechnology |  |
|  |  |  |  |  |
