# Supplementary material for: A novel long non-coding RNA from the HOXA6-HOXA5 locus facilitates colon cancer cell growth
Source: BMC Cancer. 2019 Jun 3;19:532. doi: 10.1186/s12885-019-5715-0 (PMC6547586; doi:10.1186/s12885-019-5715-0)
Supplement: Supplementary file 2 — Figure S1. Schematic representation of the primer sets used for the qPCR assay and siRNAs targeting of the HOXA6-HOXA5 locus. Primers for qPCR amplification of the indicated transcripts or genomic regions are indicated by the left/right arrows. Transcripts, HOXA5 short and HOXA5 long 1; regions, HOXA5 coding region (CR) and 3′ UTR. Targeted sequences of siRNAs #1 to #8 are indicated by the solid lines and the dashed lines for siRNAs #7 and #8 indicate the skipped sequences of the siRNAs. Figure S2. 5′-rapid amplification of cDNA ends (RACE) experiments of the HOXA6-HOXA5 locus. A. Scheme diagram of the gene-specific primers used for 5′-RACE experiment. B. Electrophoretic analysis of PCR amplification products. C. Nucleotide sequences of the PCR products. Primers used are underlined. Grey boxes indicate the junctions between different exons. M, DNA ladder marker. Figure S3. 3′-rapid amplification of cDNA ends (RACE) experiments of the HOXA6-HOXA5 locus. A. Scheme diagram of the gene-specific primers used for 3′-RACE experiment. B. Electrophoretic analysis of PCR amplification products. C. Nucleotide sequences of the PCR products. Primers used are underlined. Grey boxes indicate the junctions between different exons. M, DNA ladder marker. Figure S4. Analysis of translation potency of the HOXA5 short RNA. A. A T7 promoter-containing DNA fragments encoding full-length HOXA5 RNA, HOXA5 short RNA, or GAPDH were generated by PCR amplification and the resultant PCR products were subjected to in vitro transcription and translation assays, which included the incorporation of fluorescent lysine. The synthesized proteins were analyzed by 15% SDS-PAGE and detected using a fluoro-imaging instrument. B. The translation potency of HOXA5 short RNA was calculated using Coding-Potential Assessment Tool (CPAT) software. Sequences of the coding regions of HOXA5 and GAPDH were used as translatable sequences and that of MALAT1, known as a functional long non-coding RNA, was used as an unt [file 12885_2019_5715_MOESM2_ESM.pdf]

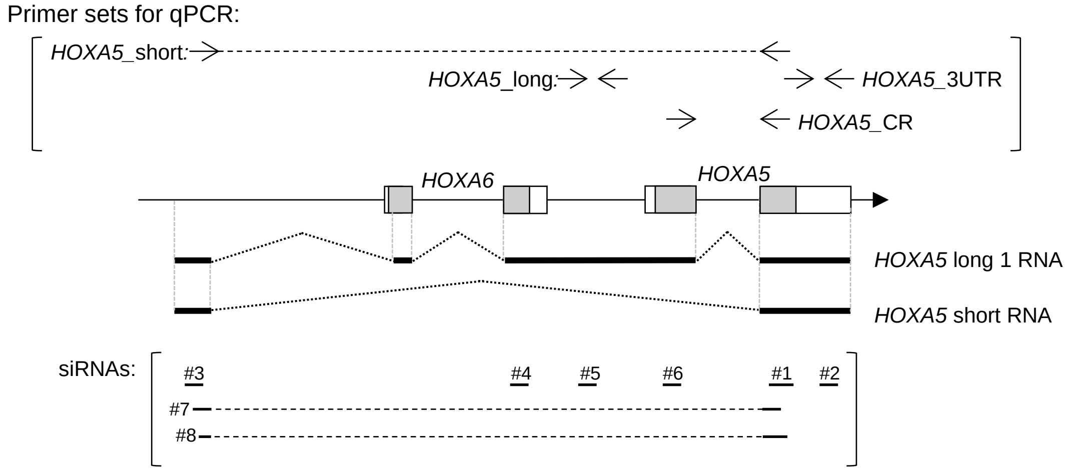

Figure S1. Schematic representation of the primer sets used for the qPCR assay and siRNAs targeting of the *HOXA6-HOXA5* locus. Primers for qPCR amplification of the indicated transcripts or genomic regions are indicated by the left/right arrows. Transcripts, *HOXA5* short and *HOXA5* long 1; regions, *HOXA5* coding region (CR) and 3' UTR. Targeted sequences of siRNAs #1 to #8 are indicated by the solid lines and the dashed lines for siRNAs #7 and #8 indicate the skipped sequences of the siRNAs.

Figure S2

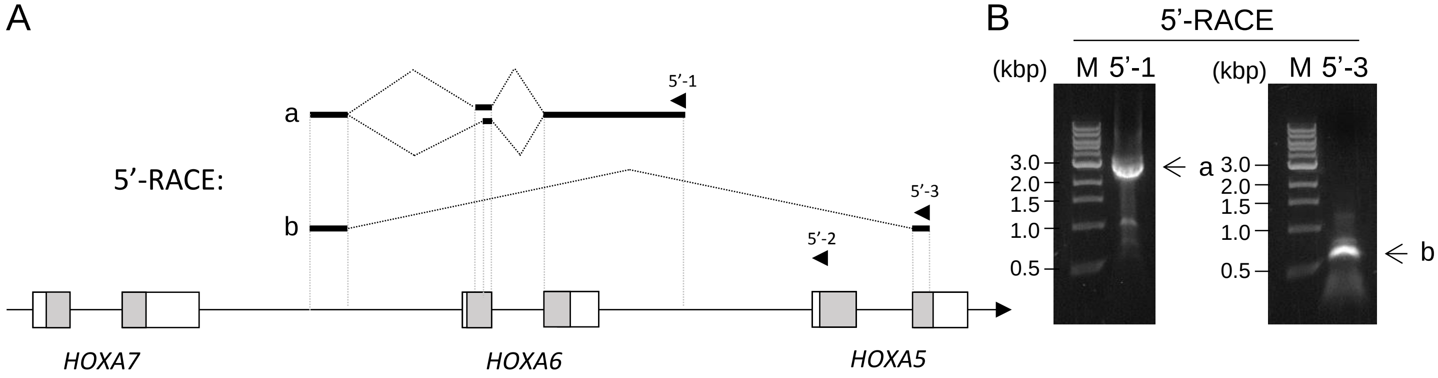

C

>5'-1 (2,182 nt): corresponding to the band "a" (partial sequence of long 1)
CTAATACGACTCACTATAGGGCAAGCAGTGGTATCAACGCAGAGTGGGACCGGCGCCAGCTGCAGCCCGCCTCTTGCAGCCTGGCGCGACTGCTGTACTGGCCCCTC
AGAGATTTCCCAGCAAGCAGGGTAGAGGGGGACGCAAGGGGACCTGGGGGGCGATCACCCAGAGCCTATATGGCCGGGAAATCTCCCCGCCTCGGCTACCCCTGACC
GGAGCTGGACGCCGTTATAGACTCGGGTCCTCCCCCACCCCCACTCCAGCACCATGGAGACTGGTTGCACAGGCCGCCTGGCACGTCATGGCTGCTTCTGTCCAAAC
TGCGCGTCCCAAGGCGTTCTGGGGGCGCACGCTGGTTCAGGGAACACTCTGGCTTTGGGATTCTGCAATCGGAGAGACTACCTGTAGTCCTGAGGCCTGTAGGCCTG
GCTGCTCTGAGGAGGATTGGCTATGGCAAAGAAGGACGGCTTTGGCTCCAGGAGAGGGCTGTGAAGGGCAGAGGCCGCACCTGTGTGCTTTGGGCTCTGGTCTGGGC
TAGCGAGCTGGCTGGACCGAGGTGGCCTTCAAGGTCCCACACCTACATACTCCCAGACCACCCTCCAGGCTGGCAGAGGTGGAGCTGTTCAATGGGCAGCCCAGGGC
AAGGGCAGACAGTGGGGCCCAGCTCGCCGTCCTGCCCCCCTTGGAGAGAAACTGAAAGTCCAACTCGGTCCTGGCCTGCAACCGGGCGTCCTACGAGTACGGGGCCT
CGTGTTTCTATTCTGATAAGGACCTCAGTGGCGCCTCGCCCTCGGGCAGTGGCAAGCAGAGGGGCCCCGGGGACTACCTGCACTTTTCTCCCGAGCAGCAGTACAAA
CCCGACAGCAGCAGCGGGCAGGGCAAAGCACTCCATGACGAAGGCGCCGACCGGAAGTACACGAGCCCGGTTTACCCTTGGATGCAGCGGATGAACTCCTGCGCGGG
TGCTGTGTATGGGAGCCATGGGCGCCGAGGCCGCCAGACCTACACGCGCTACCAGACACTGGAGCTGGAGAAGGAGTTCCACTTCAACCGCTACCTGACACGGCGCC
GCCGCATCGAGATCGCCAACGCGCTCTGCCTCACCGAGCGCCAGATCAAGATCTGGTTCCAGAACCGCCGCATGAAGTGGAAAAAGGAAAACAAGCTCATCAATTCC
ACGCAGCCCAGCGGGGAGGACTCAGAGGCAAAGGCGGGCGAGTAGATGCCTGGGCAGGGACCAGGCCAGCGCTGCAACCTCCTTCGGCTTTGCCCCCTTGCCCTCGC
CTGTTCCCCAACTTTTCTCCCCGCCTGCTCCCATCTGGGGGCTTCCGCAGCTTCAGGGGAGCCCGGAGCTTTGCAAGCGTCTGTGCATTTATTTCTTACAAAACAAA
ACAAAACAAAACAAAACAAAACAAAACAAAACAAAACCTCACACACAGCCAATACCAGCGGTGGAGCGGGGCGCACTGCACACACCCAGTCCCGCTCCACAA
GGATGCCCAAACCGGCTCCGGAGTTCTGGGGTGCGCCCTGAATGCGTCTGGGGCTCCTGTGCTAGTGTCTCTGAGCTCCCCGACCTCGGGACGCGGGGCTGCTGGGG
AGGAAAGAGCCCTGGGCCGAGCCCCGTTTCGTGTCTGGGCGTCTCTGCCTAAGTCCCTCGCCAGTAAGTCCCGAAGGGCATCATCAAATCATCTCTGGCCAGTGGAA
AGAGGGGGTCTAAGAGCTGGAGCCCGGCCTTGTCGCGGCCTCCCAAGCCGCCTCCCAGAACAGGAAGGAAGCTCGAGGAACAAAGGGGGCCCCAACAGAGCCCAGTC
TCTCGGTCCCGCGTGTGCAACCGTCAGTGGAAGAGAAGCAGCCTCAGCCGAGGCGAGTTAACCTGGGCGCGGGTGGAACATTACAGCCCGGGGTAGGCTCCCGGCTC
CGGATTCCCGCTCCCAGCCTCCGATAGCGCCCGCGTCGCCGCCACAGCAGCGTTCAGGACCCAACGAGGGGCCCAGGGCCATGGGAGCCGCCAGAGTCCTGGCTTCC
AGACGTGCCAGGGGCGCCTGCAACGCCGGGCCTCCAGCGGGGAGACTCCACTTGCCTCTCAGCTATATTTGTGATTTACTTGAGTCTTTCGGAGCCGTGGAAAATAA
CTACAGAGATACTAGGCACTGCCAAGGAATGCTTCCATTATCGCCCTCATTTACTCCTGGAGGTCTAAGCAAGACCCCAGTAACCC

>5'-1 (2,035 nt): corresponding to the band "a" (partial sequence of long 2)
CTAATACGACTCACTATAGGGCAAGCAGTGGTATCAACGCAGAGTGGGACCGGCGCCAGCTGCAGCCCGCCTCTTGCAGCCTGGCGCGACTGCTGTACTGGCCCCTC
AGAGATTTCCCAGCAAGCAGGGTAGAGGGGGACGCAAGGGGACCTGGGGGGCGATCACCCAGAGCCTATATGGCCGGGAAATCTCCCCGCCTCGGCTACCCCTGACC
GGAGCTGGACGCCGTTATAGACTCGGGTCCTCCCCCACCCCCACTCCAGCACCATGGAGACTGGTTGCACAGGCCGCCTGGCACGTCATGGCTGCTTCTGTCCAAAC
TGCGCGTCCCAAGGCGTTCTGGGGGCGCACGCTGGTTCAGGGAACACTCTGGCTTTGGGATTCTGCAATCGGAGAGACTACCTGTAGTCCTGAGGCCTGTAGGCCTG
GCTGCTCTGAGGAGGATTGGCTATGGCAAAGAAGGACGGCTTTGGCTCCAGGAGAGGGCTGTGAAGGGCAGAGGCCGCACCTGTGTGCTTTGGGCTCTGGTCTGGGC
TAGCGAGCTGGCTGGACCGAGGTGGCCTTCAAGGTCCCACACCTACATACTCCCAGACCACCCTCCAGGCTGGCAGAGGTGGAGCTGTTCAATGGGCAGCCCAGGGC
AAGGGCAGACAGTGGGGCCCAGCTCGCCGTCCTGCCCCCCTTGGAGAGAAACTGAAAGCAGTACAAACCCGACAGCAGCAGCGGGCAGGGCAAAGCACTCCATGACG
AAGGCGCCGACCGGAAGTACACGAGCCCGGTTTACCCTTGGATGCAGCGGATGAACTCCTGCGCGGGTGCTGTGTATGGGAGCCATGGGCGCCGAGGCCGCCAGACC
TACACGCGCTACCAGACACTGGAGCTGGAGAAGGAGTTCCACTTCAACCGCTACCTGACACGGCGCCGCCGCATCGAGATCGCCAACGCGCTCTGCCTCACCGAGCG
CCAGATCAAGATCTGGTTCCAGAACCGCCGCATGAAGTGGAAAAAGGAAAACAAGCTCATCAATTCCACGCAGCCCAGCGGGGAGGACTCAGAGGCAAAGGCGGGCG
AGTAGATGCCTGGGCAGGGACCAGGCCAGCGCTGCAACCTCCTTCGGCTTTGCCCCCTTGCCCTCGCCTGTTCCCCAACTTTTCTCCCCGCCTGCTCCCATCTGGGG
GCTTCCGCAGCTTCAGGGGAGCCCGGAGCTTTGCAAGCGTCTGTGCATTTATTTCTTACAAAACAAAACAAAACAAAACAAAACAAAACAAAACAAAACAAAACAAA
ACCTCACACACAGCCAATACCAGCGGTGGAGCGGGGCGCACTGCACACACCCAGTCCCGCTCCACAAGGATGCCCAAACCGGCTCCGGAGTTCTGGGGTGCGCCCTG
AATGCGTCTGGGGCTCCTGTGCTAGTGTCTCTGAGCTCCCCGACCTCGGGACGCGGGGCTGCTGGGGAGGAAAGAGCCCTGGGCCGAGCCCCGTTTCGTGTCTGGGC
GTCTCTGCCTAAGTCCCTCGCCAGTAAGTCCCGAAGGGCATCATCAAATCATCTCTGGCCAGTGGAAAGAGGGGGTCTAAGAGCTGGAGCCCGGCCTTGTCGCGGCC
TCCCAAGCCGCCTCCCAGAACAGGAAGGAAGCTCGAGGAACAAAGGGGGCCCCAACAGAGCCCAGTCTCTCGGTCCCGCGTGTGCAACCGTCAGTGGAAGAGAAGCA
GCCTCAGCCGAGGCGAGTTAACCTGGGCGCGGGTGGAACATTACAGCCCGGGGTAGGCTCCCGGCTCCGGATTCCCGCTCCCAGCCTCCGATAGCGCCCGCGTCGCC
GCCACAGCAGCGTTCAGGACCCAACGAGGGGCCCAGGGCCATGGGAGCCGCCAGAGTCCTGGCTTCCAGACGTGCCAGGGGCGCCTGCAACGCCGGGCCTCCAGCGG
GGAGACTCCACTTGCCTCTCAGCTATATTTGTGATTTACTTGAGTCTTTCGGAGCCGTGGAAAATAACTACAGAGATACTAGGCACTGCCAAGGAATGCTTCCATTA
TCGCCCTCATTTTACTCCTGGAGGTCTAAGCAAGACCCCAGTAACCC

>5'-3 (757 nt) : corresponding to the band "b" (partial sequence of short)
CTAATACGACTCACTATAGGGCAAGCAGTGGTATCAACGCAGAGTGGGACCGGCGCCAGCTGCAGCCCGCCTCTTGCAGCCTGGCGCGACTGCTGTACTGGCCCCTC
AGAGATTTCCCAGCAAGCAGGGTAGAGGGGGACGCAAGGGGACCTGGGGGGCGATCACCCAGAGCCTATATGGCCGGGAAATCTCCCCGCCTCGGCTACCCCTGACC
GGAGCTGGACGCCGTTATAGACTCGGGTCCTCCCCCACCCCCACTCCAGCACCATGGAGACTGGTTGCACAGGCCGCCTGGCACGTCATGGCTGCTTCTGTCCAAAC
TGCGCGTCCCAAGGCGTTCTGGGGGCGCACGCTGGTTCAGGGAACACTCTGGCTTTGGGATTCTGCAATCGGAGAGACTACCTGTAGTCCTGAGGCCTGTAGGCCTG
GCTGCTCTGAGGAGGATTGGCTATGGCAAAGAAGGACGGCTTTGGCTCCAGGAGAGGGCTGTGAAGGGCAGAGGCCGCACCTGTGTGCTTTGGGCTCTGGTCTGGGC
TAGCGAGCTGGCTGGACCGAGGTGGCCTTCAAGGTCCCACACCTACATACTCCCAGACCACCCTCCAGGCTGGCAGAGGTGGAGCTGTTCAATGGGCAGCCCAGGGC
AAGGGCAGACAGTGGGGCCCAGCTCGCCGTCCTGCCCCCCTTGGAGAGAAACTGAAAGACAACATAGGCGGCCCGGAAGGCAAAAGGGCCCGGACGGCCTACACGCG
CTACCAGACCCTGGAGCTGGAGAAGGAGTTCCACTTCAACCGTTACCTGACCC

Figure S2. 5'-rapid amplification of cDNA ends (RACE) experiments of the *HOXA6-HOXA5* locus. A. Scheme diagram of the gene-specific primers used for 5'-RACE experiment. **B.** Electrophoretic analysis of PCR amplification products. **C.** Nucleotide sequences of the PCR products. Primers used are underlined. Grey boxes indicate the junctions between different exons. M, DNA ladder marker.

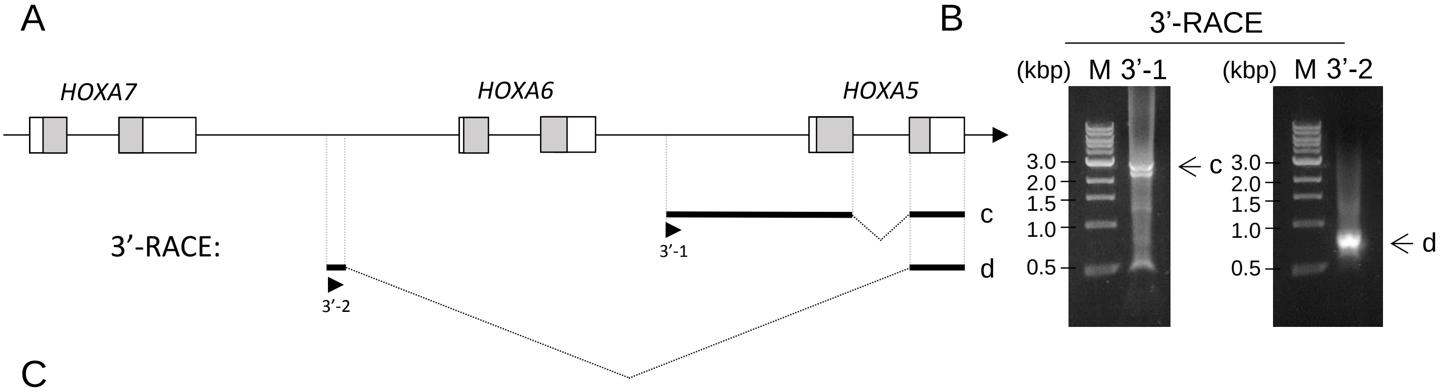

C

>3'-1 (2,792 nt): corresponding to the band "c" (partial sequence of long 1 and 2)
GACTCCACTTGCCTCTCAGCTATATTTGTGATTTACTTGAGTCTTTCGGAGCCGTGGAAAATAACTACAGAGATACTAGGCACTGCCAAGGAATGCTTCCATTATCG
CCCTCATTTTACTCCTGGAGGTCTAAGCAAGACCCCAGTAACCCGCCCCACGGATCCCCAGACCCCAGGCAGCCTCTGTGGCTGCAGGGAGCCTGGGCCCACTCGCT
GGGTTCAAGGAGAACCCTCCGACTTGGGGCCCGGCTGGCTCGAGGAACCGGACCCTGTGATGAAGATTTTCCAGGCTGGATACGTAAGCAAATCAAATCGCAAACTA
ATGACACGAAAACCATATTCACACGAAAGAAAAATCGACTACGGTTATAAAAGTGTATGGAATTTGACCTCGCCTTGGAGAAACACTACACAAAAGCTATCTTTAAT
AGACGCCTGTCATTTAAGAGCGGCAGGGACACTGTCCCTCATGCGCTCGCAAAAACAGAGCCGTAATTGTAGCTGCTGCTGCTGCGGGCAGGATTTATTTCTCCAAT
TGGCTAAATGGCTTTCCCCCTTCCCCGAAGGTGATATCTGTATTTTCAAAATTCAGAGCTGCTGGCAGGACGGTCAGTACCGACCCCAACCTCGACACAAAAATAAG
AGGGGCTGCAAAACGGGGGAAATAAAGTTGTTGTAAATAAAATGCAAGTCACCACCTCCCCCCAATCCTCTGCATCCTCGCCGGGCGCGCGATCGGCAGCTGACGGC
CTAACAATTGGTACATCCTAATGGAACTGCGAGGGAAATGCAATAATTTTGCCATAATGGGCTGTAACCTCAATTCGACCCCGGCCCTTGCAGCCCCCGGTCGGAAG
CTGGGCGATGAGCCCTGCCTCCAGCGGGTGGCGCTCGAGTCCGGCTGAACGGCGGCAACTGGCGGCGGGCACGCGCCCGGGGCGCGCGCGCCACCCCCCTCGCCTCC
ACCCAACTCCCCTATTAGTGCACGAGTTTACCTCTAGAGGTCATCAGGCAGGATTTACGACTGGACAACAAAAGCACGTGATTCGAAGTCGTACCCCATATTTGGGT
GCCTACGTAGGAGGGAACCAAGTACATGTCCCAGTCATTTCCATAATTCATCATAAATTGTGCAAGGGTGCTATAGACGCACAAACGACCGCGAGCCACAAATCAAG
CACACATATCAAAAAACAAATGAGCTCTTATTTTGTAAACTCATTTTGCGGTCGCTATCCAAATGGCCCGGACTACCAGTTGCATAATTATGGAGATCATAGTTCCG
TGAGCGAGCAATTCAGGGACTCGGCGAGCATGCACTCCGGCAGGTACGGCTACGGCTACAATGGCATGGATCTCAGCGTCGGCCGCTCGGGCTCCGGCCACTTTGGC
TCCGGAGAGCGCGCCCGCAGCTACGCTGCCAGCGCCAGCGCGGCGCCCGCCGAGCCCAGGTACAGCCAGCCGGCCACGTCCACGCACTCTCCTCAGCCCGATCCGCT
GCCCTGCTCCGCCGTGGCCCCCTCGCCCGGCAGCGACAGCCACCACGGCGGGAAAAACTCCCTAAGCAACTCCAGCGGCGCCTCGGCCGACGCCGGCAGCACCCACA
TCAGCAGCAGAGAGGGGGTTGGCACGGCGTCCGGAGCCGAGGAGGACGCCCCTGCCAGCAGCGAGCAGGCGAGTGCGCAGAGCGAGCCGAGCCCGGCGCCGCCCGCC
CAACCCCAGATCTACCCCTGGATGCGCAAGCTGCACATAAGTCATGACAACATAGGCGGCCCGGAAGGCAAAAGGGCCCGGACGGCCTACACGCGCTACCAGACCCT
GGAGCTGGAGAAGGAGTTCCACTTCAACCGTTACCTGACCCGCAGAAGGAGGATTGAAATAGCACATGCTCTTTGCCTCTCCGAGAGACAAATTAAAATCTGGTTCC
AAAACCGGAGAATGAAGTGGAAAAAAGATAATAAGCTGAAAAGCATGAGCATGGCCGCGGCAGGAGGGGCCTTCCGTCCCTGAGTATCTGAGCGTTTAAAGTACTGA
GCAGTATTAGCGGATCCCGCGTAGTGTCAGTACTAAGGTGACTTTCTGAAACTCCCTTGTGTTCCTTCTGTGAAGAAGCCCTGTTCTCGTTGCCCTAATTCATCTTT
TAATCATGAGCCTGTTTATTGCCATTATAGCGCCTGTATAAGTAGATCTGCTTTCTGTTCATCTCTTTGTCCTGAATGGCTTTGTCTTGAAAAAAAATAGATGTTTT
AACTTATTTATATGAAGCAAGCTGTGTTACTTGAAGTAACTATAACAAAAAAAGAAAAGAGAAAAAAAAACACACAAAAAGTCCCCCTTCAATCTCGTTTAGTGCCA
ATGTTGTGTGTTGCACTCAAGTTGTTTAACTGTGCATGTGCGTGGAAGTGTTCCTGTCTCAATAGCTCCAAGCTGTTAAAGATATTTTTATTCAAACTACCTATATT
CCTTGTGTAATTAATGCTGTTGTAGAGGTGACTTGATGAGACACAACTTGTTCGACGTGTAGTGACTAGTGACTCTGTGATGAAAACTGTGACTCCAAGCGGTGTGT
CCCTGCGTGCCTTTATAGGACCCTTTGCACGAACTCTGGAAGTGGCTCTTATAAGCGCAGCTTCAGTGATGTATGTTTTTGTGAACAAAGTTACAAATATTGTCCAA
GTCTGGCTGTTTTAAGCAAACTGTGATCAGCTTTTTTTTTTTTTTTTTTTTTTTTGTATTTGTTTTTAAGGAAAAAATACTGACTGGAACAAAAAATAAACTTTCTA
TTGTAAGTTCCAAAAAAAAAAAAAAAAAAAGTACTCTGCGTTGATACCACTGCTTGCCCTATAGTGAGTCGTATTAG

>3'-2 (1,124 nt): corresponding to the band "d" (partial sequence of short)
GAGGTGGAGCTGTTCAATGGGCAGCCCAGGGCAAGGGCAGACAGTGGGGCCCAGCTCGCCGTCCTGCCCCCCTTGGAGAGAAACTGAAAGACAACATAGGCGGCCCG
GAAGGCAAAAGGGCCCGGACGGCCTACACGCGCTACCAGACCCTGGAGCTGGAGAAGGAGTTCCACTTCAACCGTTACCTGACCCGCAGAAGGAGGATTGAAATAGC
ACATGCTCTTTGCCTCTCCGAGAGACAAATTAAAATCTGGTTCCAAAACCGGAGAATGAAGTGGAAAAAAGATAATAAGCTGAAAAGCATGAGCATGGCCGCGGCAG
GAGGGGCCTTCCGTCCCTGAGTATCTGAGCGTTTAAAGTACTGAGCAGTATTAGCGGATCCCGCGTAGTGTCAGTACTAAGGTGACTTTCTGAAACTCCCTTGTGTT
CCTTCTGTGAAGAAGCCCTGTTCTCGTTGCCCTAATTCATCTTTTAATCATGAGCCTGTTTATTGCCATTATAGCGCCTGTATAAGTAGATCTGCTTTCTGTTCATC
TCTTTGTCCTGAATGGCTTTGTCTTGAAAAAAAATAGATGTTTTAACTTATTTATATGAAGCAAGCTGTGTTACTTGAAGTAACTATAACAAAAAAAGAAAAGAGAA
AAAAAAACACACAAAAAGTCCCCCTTCAATCTCGTTTAGTGCCAATGTTGTGTGTTGCACTCAAGTTGTTTAACTGTGCATGTGCGTGGAAGTGTTCCTGTCTCAAT
AGCTCCAAGCTGTTAAAGATATTTTTATTCAAACTACCTATATTCCTTGTGTAATTAATGCTGTTGTAGAGGTGACTTGATGAGACACAACTTGTTCGACGTGTAGT
GACTAGTGACTCTGTGATGAAAACTGTGACTCCAAGCGGTGTGTCCCTGCGTGCCTTTATAGGACCCTTTGCACGAACTCTGGAAGTGGCTCTTATAAGCGCAGCTT
CAGTGATGTATGTTTTTGTGAACAAAGTTACAAATATTGTCCAAGTCTGGCTGTTTTAAGCAAACTGTGATCAGCTTTTTTTTTTTTTTTTTTTTTTTTGTATTTGT
TTTTAAGGAAAAAATACTGACTGGAACAAAAAATAAACTTTCTATTGTAAGTTCCAAAAAAAAAAAAAAAAAAAAAAAGTACTCTGCGTTGATACCACTGCTTGCCC
TATAGTGAGTCGTATTAG

Figure S3. 3'-rapid amplification of cDNA ends (RACE) experiments of the *HOXA6-HOXA5* locus. A. Scheme diagram of the gene-specific primers used for 3'-RACE experiment. **B.** Electrophoretic analysis of PCR amplification products. **C.** Nucleotide sequences of the PCR products. Primers used are underlined. Grey boxes indicate the junctions between different exons. M, DNA ladder marker.

Figure S4

A

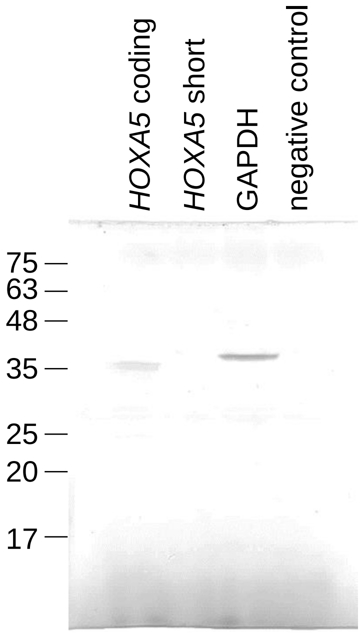

B

RNA Name	Coding Probability	Coding Label
HOXA5 short	0.0448	no
HOXA5 coding	0.997	yes
MALAT1	0.0142	no
GAPDH	0.999	yes

Figure S4. Analysis of translation potency of the *HOXA5* short RNA. A. A T7 promoter-containing DNA fragments encoding full-length HOXA5 RNA, *HOXA5* short RNA, or GAPDH were generated by PCR amplification and the resultant PCR products were subjected to *in vitro* transcription and translation assays, which included the incorporation of fluorescent lysine. The synthesized proteins were analyzed by 15% SDS-PAGE and detected using a fluoro-imaging instrument. **B.** The translation potency of *HOXA5* short RNA was calculated using Coding-Potential Assessment Tool (CPAT) software. Sequences of the coding regions of *HOXA5* and *GAPDH* were used as translatable sequences and that of *MALAT1,* known as a functional long non-coding RNA, was used as an untranslatable sequence.

```
       -74                       -53  TATA box  -39
Human: ACTGCGGCTGCCGCGGGCCGATTATTTATTGCGACCGTGCTGG-CGCTCGCTG
Horse: ACTGCGGCTGCCGCGGGCAGATTATTTATTGCGACCGCGCTAG-CGCTCGCTG
Cow:   ACTGCGGCTGCCGCGGGCAGATAATTTATTGCGACCGTGCCGGGCGCTCGCTG
Mouse: ACTGCGGCTGCCGCAGGCGGATTATTTATTGCGACCGAACAGGGAGCTCGCTG
Rat:   ACTGCGGCTGCCGCAGGCGGATTATTTATTGCGACCGAGCCGTGAGCTCGCTG

                            TSS
                                                +20
Human: CTGCCACGCCGTGGCCGTCGCCGGGACCGGCGCCAGCTGCAG
Horse: CTGCCACGCTGTGGCCGTCACCGGGACCGACGCCAGCCACAG
Cow:   CTGCCACGCTGTGGCCATCTCCGGAACCGACGCCAATAGCAG
Mouse: CTGCCACGCTGTGGCCGTCGCCGGGACCGGCGCCAGCAGCGT
Rat:   CTGCCACGCTGTGGCCGTCGCCGGGACCGGCGCCAGCAGCGG
```

Figure S5. Evolutionary conserved sequences of a transcriptional start site of the *HOXA5 short RNA.*
Sequence alignment of the upstream sequences of a transcriptional start site (TSS) in *HOXA5* short RNA indicates the presence of a consensus TATA box and a TSS in most species.

Figure S6

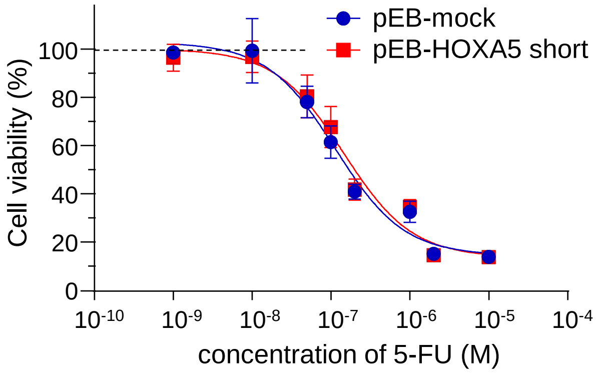

Figure S6. Intrinsic chemoresistance to 5-FU in HOXA5 short RNA expressing HCT116 cells. The cell viability of pEB-HOXA5 short or pEB-mock HCT116 cells was determined by Cell Count Reagent *SF* after treatment with increasing doses of 5-FU for 48 h.

Figure S7

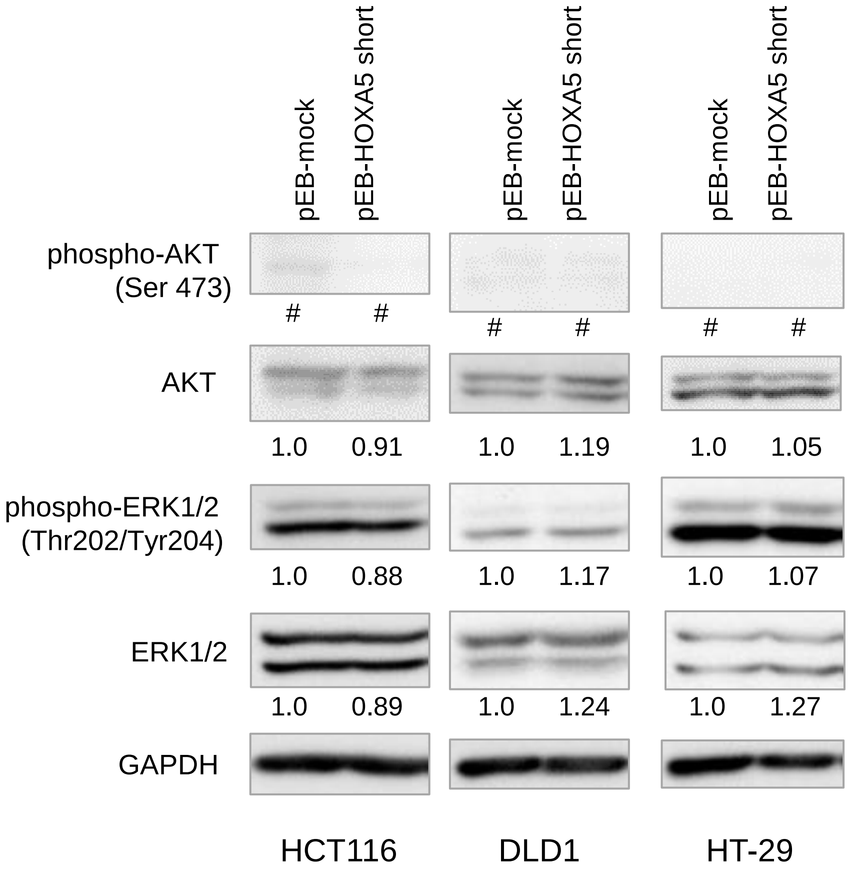

Figure S7. Effects of *HOXA5* short RNA on AKT and ERK activation. Protein levels of phosphorylated AKT (Ser473; #9271, Cell Signaling Tech.), total AKT (#9272, Cell Signaling Tech.), phosphorylated ERK1/2 (#9101, Cell Signaling Tech.) and total ERK1/2 (#9102, Cell Signaling Tech.) were measured by western blot analysis. GAPDH levels were used as an endogenous quantitative control. The level of phospho-AKT, phosphor-ERK1/2, AKT or ERK1/2 band relative to that of GAPDH was quantitatively analyzed by densitometry. #: The band corresponding to phospho-AKT was not sufficiently detected for densitometry analyses.
